# Accurate EDM Calibration of a Digital Twin for a Seven-Axis Robotic EDM System and 3D Offline Cutting Path †

**DOI:** 10.3390/mi16080892

**Published:** 2025-07-31

**Authors:** Sergio Tadeu de Almeida, John P. T. Mo, Cees Bil, Songlin Ding, Chi-Tsun Cheng

**Affiliations:** School of Aerospace, Mechanical and Manufacturing Engineering, RMIT University, East Campus, Melbourne, VIC 3083, Australia; john.mo@rmit.edu.au (J.P.T.M.); cees.bil@rmit.edu.au (C.B.); songlin.ding@rmit.edu.au (S.D.); ben.cheng@rmit.edu.au (C.-T.C.)

**Keywords:** electric discharge machining, robotic EDM, digital twin, robotic calibration, offline cutting path programming

## Abstract

The increasing utilization of hard-to-cut materials in high-performance sectors such as aerospace and defense has pushed manufacturing systems to be flexible in processing large workpieces with a wide range of materials while also delivering high precision. Recent studies have highlighted the potential of integrating industrial robots (IRs) with electric discharge machining (EDM) to create a non-contact, low-force manufacturing platform, particularly suited for the accurate machining of hard-to-cut materials into complex and large-scale monolithic components. In response to this potential, a novel robotic EDM system has been developed. However, the manual programming and control of such a convoluted system present a significant challenge, often leading to inefficiencies and increased error rates, creating a scenario where the EDM process becomes unfeasible. To enhance the industrial applicability of this robotic EDM technology, this study focuses on a novel methodology to develop and validate a digital twin (DT) of the physical robotic EDM system. The digital twin functions as a virtual experimental environment for tool motion, effectively addressing the challenges posed by collisions and kinematic singularities inherent in the physical system, yet with proven 20-micron EDM gap accuracy. Furthermore, it facilitates a CNC-like, user-friendly offline programming framework for robotic EDM cutting path generation.

## 1. Introduction

High-end industries such as defense and aerospace rely on accurately shaped exotic materials. However, machining hard-to-cut materials imposes significant forces, in particular while using traditional machining techniques. Subsequently, electric discharge machining (EDM) has been widely adopted to cut any material, independently of its hardness, with at least 0.01 S/cm of electric conductivity. The EDM cutting principle consists of an electrode continuously moved towards the workpiece till a particular gap offers an optimal level of electric discharges to be sustained along with a programmed shaping cutting path. Such discharges will then gradually melt portions of the workpiece surface while the programmed path of the electrode shapes the desired geometry. Up to the present day, to control the electrode path, EDM is configured on computer numerically controlled (CNC) machines. However, CNC machines are designed for stiffness so that the traditional high forces of conventional processes can be managed accordingly. However, such rigid bed design often results in a limited envelope and reduced number of axes, ultimately leading to a restrictive plan for EDM manufacturing rules, which impose workpieces to be segmented into multiple machining steps, compromising accuracy, timeline, and costs.

Traditional machining techniques using six-axis industrial robots (IRs) have been extensively investigated to machine monolithic and complex workpieces. However, due to the intrinsic robotic arm design, IRs lack stiffness and cannot hold heavy loads for a significant time, nor can they cope with the high forces necessary to machine hard and exotic materials using traditional machining techniques. As a result, IR machining of hard-to-cut materials with conventional techniques often results in poor surface finishing and a lack of precision, so IRs are still confined to machining soft materials. To overcome this obstacle, recent research efforts have focused on solving IR vibration [1] by combining lightweight EDM end-effectors into IRs [2]. What makes EDM a suitable machining technique to combine into a robot is that, in EDM, there is no physical contact between the electrode and the workpiece, and the cutting process is conducted with nearly no forces, so that prohibitive robot arm vibrations can be avoided.

Programming cutting path machining strategies for traditional processes is deterministic and consists of defining the paths and shapes to be machined, such as roughing, finishing, and cornering strategies, which influence the performance and accuracy of the cutting process. In brief, the cutting path programs consist of deriving from the 3D CAD file of the workpiece the 3D file of the necessary stock material. Next, depending on the workpiece geometry, desired finishing surface, and tolerances, either one cutting path or a combination of various ordered and overlapped cutting paths will be calculated. For each of these cutting path programs, several process parameters must be defined, including but not limited to the cutting tools available and selected, tool speed and feeds, toolpath strategy, stepover and stepdown, cutting direction, entry and exit points, material properties, coolant and lubrication, stock allowance, rapid moves and retract heights, safety, and error checking. In addition to these briefly described cutting path programming considerations, EDM is a non-traditional machining technique where several non-trivial additional cutting path process parameters must also be considered; a brief, non-exhaustive, contextualized list of these is presented below:

- **Discharge Current:** The intensity of the electrical current passing through the workpiece and electrode determines the material removal rate and the size of the spark created. In context, this parameter will define the reaction forces acting on the cutting tool electrode [3].
- **Pulse Duration (On-Time):** This is the duration for which the current is applied during each pulse. It influences the amount of material removed and the heat generated during the spark. In context, together with the discharge current, these parameters will define the feasible feed rate for the cutting path program [4].
- **Pulse Frequency (Off-Time):** This refers to the interval between successive electrical discharges. This parameter affects the efficiency of material removal and the overall machining time. In context, along with the pulse duration and discharge current, these parameters will define the reaction force and the risky vibration on commonly low-stiffness robotic arms [3].
- **Pulse Gap Distance:** The distance where the voltage between the electrode and the workpiece tends to result in optimal material rate removal and a stable process. In context, this value varies from 5 to 100 μm and must be added to the electrode’s surface to define the cutting tool’s effective size and geometry [5].
- **Electrode Material:** The material used for the electrode impacts the machining rate, wear ratio, and surface finish. Common materials include copper, graphite, and tungsten. In context, depending on the material and amount of material to be removed, compensation movements must be added to the cutting path program to consider electrode wear and deliver an accurate cut [3].
- **Dielectric Fluid:** The type and condition of the dielectric (usually deionized water or oil) impact the flushing of debris, the cooling of the workpiece, and the efficiency of the process. In context, cutting path programs could include pre-defined retract movements with the intention of better flushing of debris for a more stable operation and improved surface roughness. It is important to note that for complex geometries, such as uneven surfaces, this retract movement is not a simplistic linear movement but a backwards movement consisting of reversing the programmed cutting path for a pre-defined length, speed, and time [3].

Based on the above-described aspects of both (i) cutting path programming and (ii) contextualized EDM process parameters, it becomes evident that even for simple prismatic linear 3D workpieces, nearly manual robot programming techniques such as teaching would be time-consuming and expensive, and we could almost be sure that the optimal solution would not be explored. At this point, it is crucial to clarify that the cutting path does not consist in controlling the EDM process, which is, to put it simply, the one responsible for defining how fast the path is executed, finished, or interrupted [4].

Therefore, the present research aims to fill the gap in the cutting path programming research by developing a robotic EDM digital twin (DT) that can serve as a tool for complex multiple-axis programming, making available the best machining path strategies while coping with IR collision and singularities and EDM high-calibration accuracy needs. Therefore, this article is a revised and expanded version of a paper entitled “A Novel Robotic EDM Digital Twin for Offline Cutting-Path Programming” [6], which was presented at the 30th ISTE International Conference on Transdisciplinary Engineering, Hua Hin Cha Am, Thailand, 11–14 July 2023. Its ultimate contribution is to deliver an intuitive CAD-to-CNC-like coding approach for offline robotic EDM cutting path programming, approximating robotic EDM to real-world CNC industrial applications. The paper is organized as follows: Section 2 presents a literature review on DT within the robotic and machining context. Section 3 describes the methodology to be developed and sets the solution. Section 4 presents the validated results, while Section 5 provides concluding remarks.

## 2. Literature Review on Digital Twins in the Manufacturing and Machining Context

The first record of the “twin” concept originated in the early 1970s with NASA’s Apollo 13 project, where two identical spacecraft were created. One stayed on Earth, serving as a mirror for the mission craft. The Earth-bound twin aided in simulation verification, training, reflecting, and predicting the mission craft’s status during execution. In 2003, Dr Michael Grieves introduced the idea of a “*virtual digital representation equivalent to a physical product*”, first in product lifecycle management, which later evolved to the term “digital twin” in 2011 [7]. The concept gained acceptance, transforming the Apollo twin idea into a virtual space, connecting physical and virtual realms by data exchange, creating corresponding digital twins for products, factories, and complex systems, and establishing a rich and dynamic virtual space.

### 2.1. The Concept of a Digital Twin

The current literature includes various standards and digital twins in the context of smart manufacturing. These standards include IEC TS 62832 [8], IEEE P2806 [9], IPC 2551 [8], DIN SPEC (Asset Administration Shell), and ISO 23247 [10]. They define frameworks, architectures, and guidelines for establishing and maintaining digital representations of production systems, supporting data exchange, interoperability, and the implementation of DTs in industrial applications.

Since DT is a relatively new concept, different definitions of DT exist, while the ISO 23247 [10] defines a DT as “*a fit-for-purpose digital representation of an observable manufacturing element (OME) with synchronization between with its digital representation.*” However, when it comes to DT types, the literature is not unanimous but can be summarized as follows:

- **Digital Twin Prototype (DTP) and Digital Twin Instance (DTI)** [11], where DTP involves creating a virtual version for commissioning before making the physical twin, while DTI is connected to its physical counterpart throughout its lifecycle, enabling bidirectional data flow.
- **Level of Integration** [12], where a DT is called a **Digital Model** when it requires manual data exchange between the physical and digital objects, reflecting changes in one but not the other.
- **Application** [7,13], including Product DT, Production DT, and Performance DT, used for prototyping, process validation, and decision-making based on data analysis.
- **Hierarchy** [14], including Unit Level, System Level, and System of Systems (SoS) Level, classifies DTs based on the magnitude involved in manufacturing, from the smallest unit to interconnected systems.
- **Level of Maturity and Sophistication** [15] describes Partial DT, Clone DT, Augmented DT, Pre-Digital Twin, Digital Twin, Adaptive Digital Twin, and Intelligent Digital Twin [16]. These classifications rely on sophistication levels of data acquisition, ranging from basic data points to advanced capabilities like machine learning and real-time autonomous decision-making.

**Figure 1:**
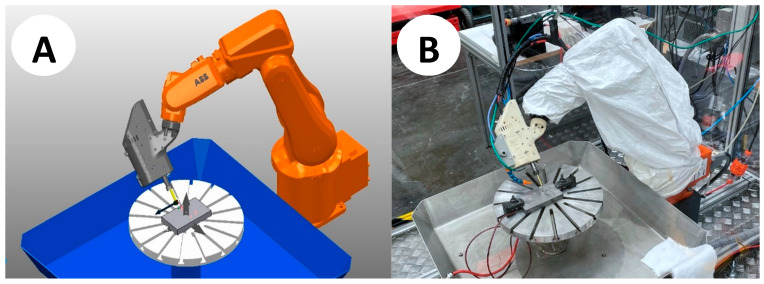
(**A**) Digital twin vs. (**B**) robotic MEDM apparatus at a 45° corner cut.

Therefore, the level of sophistication of a digital twin depends on the objectives it aims to achieve. It is worth noting that in the present study, the proposed DT started as a DTP that evolved to a level of integration with manual data exchange and a hierarchy that is defined by the physical robot assembly that carries manufacturing deviations reflected in its digital version. A representation of both physical and digital twins applied to this paper can be seen in Figure 1.

### 2.2. Applications of Digital Twins

Many other contemporary researchers have provided DT case studies with suggestions on characteristics, classifications, and types. The definition of digital twins is still open for debate. Aiming to address this gap, Singh and Fuenmayor [17] conducted a comprehensive review of DT creation, integration levels, applications, hierarchy, and maturity levels. Uhlemann and Lehmann [18] developed a DT concept for Small Manufacturing Enterprises (SMEs) focusing on data acquisition and analytics to predict service degradation performance and trends. The methods for data acquisition cope with the linkage of isolated solutions to an overall system to achieve near-real-time production control applications with no machine data, which allows for complete know-how protection and inherent data security. Altintas and Brecher [19] introduced the virtual machine tool concept while developing a DT as a virtual prototyping of machine tools, including Multi-Body Simulations with Finite Element Modeling (FEM) of the machine tool components, their kinematics, and cutting path process.

Regarding specific studies using DTs for machining purposes, Huo and Cheng [20] proposed a DT with an integrated dynamic design and modeling approach for the development of a micro-milling machine, including the micro-milling process and the dynamic models of the sub-components of the device, to measure the impact of their design on the accuracy of the process to optimize the design. Guodong [21] explored the ISO 23247 [10] Digital Twin Framework for Manufacturing to build a DT for the virtual commissioning of a CNC machine. They connected a real controller to the DT to test and optimize control strategies, control parameters, and cutting path programs, allowing for the simulation, detection, and fixing of problems before the real commissioning.

Three-dimensional models are the cornerstone of DTs. However, 3D models for legacy machines and shop floor architectures are not always available. To fill this gap, Sommer and Stjepandić [22] developed an automated scanning method to capture and store data for model representations within the often-complex SME environments, including repetitive geometries, irregular-shaped objects, and noisy measurements. To explore the full potential of sophisticated DTs, Ghosh and Ullah [23] developed an automated computerized system to build what can be classified as an Intelligent Digital Twin [16] capable of performing machining tool monitoring and troubleshooting. The system is developed using the Java™ platform, considering Input, Modeling, Simulation, Validation, and Output. The system ensures data mining for the machining process, machine tools, machining conditions, and sensors, allowing various machine learning methods to be used.

Hence, although the technologies supporting the development of digital twins are available, not all tools and methods are readily deployable to create a useful digital twin. Substantial effort and investment are anticipated in their implementation.

### 2.3. Digital Twins in Robotic Machining 

Aiming to address the difficulties of robotic cutting path programming magnified by hard-to-cut materials, a European collaborative project named “Hard Material Small-Batch Industrial Machining Robot (HEPHESTOS)” was launched in September 2012. The project was intended to provide standard industrial robots with advanced machining, cutting path programming techniques, and real-time control. However, according to Schreck and Surdilovic [24], despite great attention from advanced industries, the solution proved to be too expensive.

Aiming to bridge CNC-like programming capabilities and robotic machining, in 2013 the European Commission launched the COMET research project to develop innovative robot-based machining systems [25]. As a result, a new version of PowerMill was added, with a robot plug-in that combines a CNC toolpath programming platform with a DT-building environment for robotic offline programming, including cutting path simulation, analysis, calibration, and optimization tools. Once approved, the optimized cutting path can be exported as programs in the robot’s native language.

Since then, many other researchers have contributed to this line of research. Garnier and Subrin [26] used Matlab 2023a to build a so-called Prototype DT to investigate the stability and performance of the trajectory of mobile machining robots. As a result, a simple DT platform has been developed to compare simulation and theoretical results, where a good correlation has been achieved. Stavropoulos and Manitaras [27] developed a virtual commissioning DT of a robotic machining tool that was not installed or connected to a real controller. They adopted a Multi-Body Simulation (MBS) to capture the force dynamic behavior and the specific force coefficients required for the adopted robot model during the machining process or the real robot. They found that a DT is crucial to defining the boundary conditions of the simulation, leading to a reliable virtual prototype of the whole system and reducing the need for physical prototyping and trial and error.

Using robot machining for cutting path planning in the real world is time-consuming and poses safety risks, while 3D monitoring of the milling process is complex. To overcome these obstacles, Zhu and Lin [28] used the Unity3D platform to design and implement a DT robotic milling simulation and visualization monitoring system. Based on virtual–real mapping technology, the system has bidirectional communication between virtual and physical twins to execute the simulation and map the robot cutting path, including monitoring the material removal rate in real time.

Recently, Ye and Yang [29] used a DT approach to propose a machining performance index to optimize workpiece location using Particle Swarm Optimization (PSO) and improve the contour accuracy of conventional robotic milling. The cutting forces and deformation errors are simulated along the milling cutting path according to the robot DT stiffness model, and the contour errors are evaluated according to the deformation errors. Based on experiments, the model proved good accuracy with the robotic OME.

Based on the brief literature review, it can be concluded that cutting path programming of industrial robots is a challenging task which has been the object of relevant research in the last decade, which suggests a DT approach is a promising solution. Nonetheless, a gap in research aiming to solve the problems within non-conventional EDM techniques exists. To this end, this paper presents an approach for developing and integrating a digital twin to facilitate a user-friendly offline programming framework for robotic EDM cutting path generation.

## 3. Development of a Robotic EDM Digital Twin

A working robotic EDM system was developed in a previous research study [30]. The physical robotic EDM system has options for machining using either EDM milling or EDM wire-cut technologies. Since the hardware system configuration data is readily available, it is used as the target twinning system in this investigation.

By following the ISO framework in convergence to specific EDM process needs, a practical DT for robotic–EDM offline cutting path programming must ensure the following abilities: (i) converting workpiece 3D models into cutting path trajectories, (ii) performing offline programming in a native robotic language, (iii) conducting kinematic simulations that detect and avoid trajectory collision and singularities, and (iv) allowing for reusing mature and broadly accepted cutting path machining technics.

### 3.1. System Development Plan

**Figure 2:**
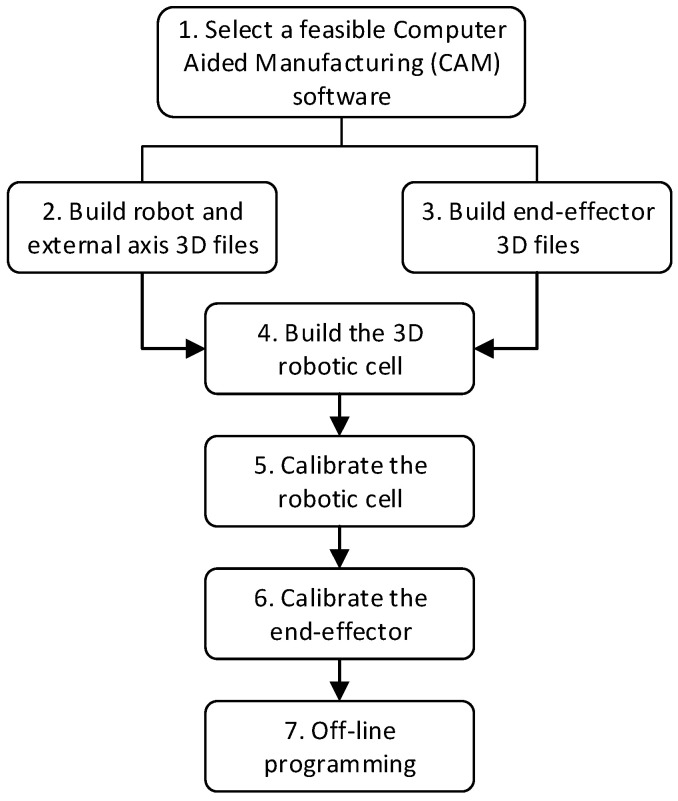
Main steps to achieve offline EDM robotic cutting path program control.

To develop a system that possesses the above four abilities, a number of steps are required to build the necessary functions into the system. Figure 2 summarizes the methodology workflow to build the DT.

Next, the following sections will develop each of the methodology steps of Figure 2.

### 3.2. Selecting Feasible CAM Software

The robotic EDM cell consists of a 7-axis system composed of a 6-axis robot model, ABB-IRB120, to which a customized robotic-controlled rotating table model ABB-MTD250 is added as the 7th axis. Creating cutting path strategies for a 7-axis robotic system is not trivial. Even for mature traditional machining techniques such as 5-axis CNC milling, converting the workpiece’s three-dimensional CAD shape into a sequence of overlayed cutting paths will demand the use of built-to-suit computer-aided manufacturing (CAM).

As reviewed in the literature, diverse types of CAM software currently exist, while PowerMill [26] stands out since it includes an extensive library of mature and successful toolpath machining strategies. Most importantly, PowerMill has a library of robot files for popular manufacturers’ most-used models. Still, if a particular model is absent, which is the case in this research, the software allows for importing and creating customized robot DTs.

### 3.3. Building the Robot’s External 3D Models

Accurate 3D models of the robot are essential. For PowerMill to perform kinematic analysis, the CAD input should be a solid model in Standard for the Exchange of Product model data (STEP) or Initial Graphics Exchange Specification (IGES) surface file format.

**Figure 3:**
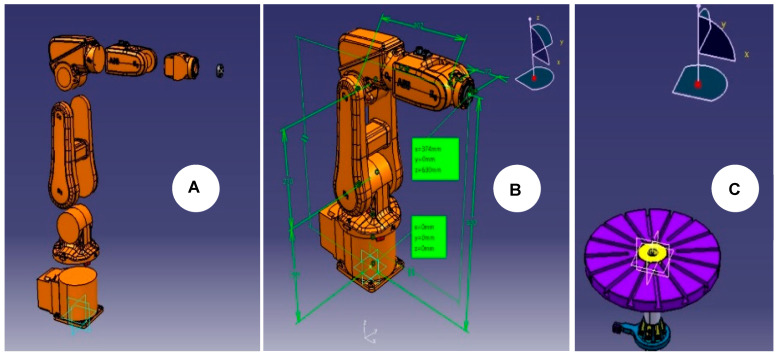
CAD file preparation: (**A**,**B**) the robot arm and (**C**) the 7th axis. (Note: Figure 1 and Figure 6 shows the combined components).

In addition, the robot manual retrieves information about the axes’ limits, lengths, and orientations. The seven 3D models acquired in Figure 3A, of which the robot arm is composed, are imported into the CATIA CAD modeler to assemble and verify that all the models are positioned at the robot’s home position and oriented according to the manual. Also, using CATIA will allow us to reuse the end-effector files and to make any necessary corrections on importing external file formats from the robot manufacturer in order to ultimately export the files to PowerMill using the ideal solid CAD type of step.

It is essential to highlight that not all robot manufacturers use the same orientation. Thus, for PowerMill to run an ABB simulation, it is crucial to position the robot base in the *XYZ* zero coordinates. As a result, Figure 3B presents the assembly of the IRB120 at its home position, with the robot base at the *XYZ* zero coordinates and the end-effector tool attachment at *X* (374), *Y* (0.0), and *Z* (630), ready to be exported in step format. Lastly, the 7th axis is a rotating table, and Figure 3C shows the 3D model positioned accordingly.

### 3.4. Build the End-Effectors 3D Models

**Figure 4:**
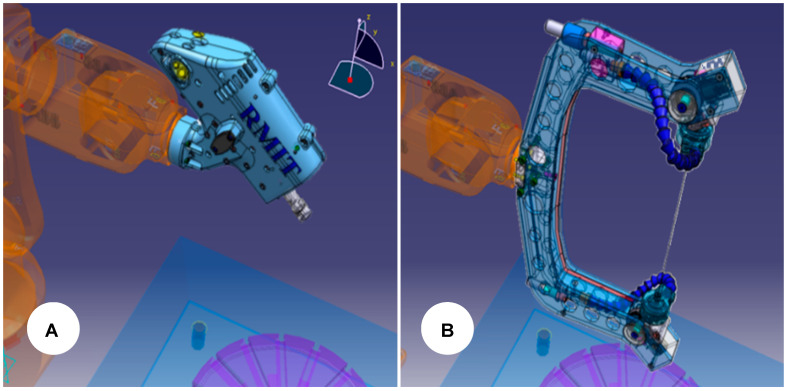
CAD file preparation of milling (**A**) and wire (**B**) end-effectors for offline programming.

As previously demonstrated, wire [5] and milling [3] EDM are the most promising EDM variants to install on an IR and they will be adopted here. Thus, using CATIA, the end-effectors are assembled following the ABB orientation. This strategy is advantageous since it will automatically position the 3D files later imported into PowerMill, as shown in Figure 4.

### 3.5. Building the 3D Robotic EDM Cell Environment

Avoiding collisions is critical for the robot and the EDM pulse. In this case, in the static 3D geometry of the robotic EDM cell, only the dielectric collector is subject to collisions; note that additional files, like the robotic cell cage, can be included for enhanced collision check. The first step in creating the robotic cell is to prepare a folder structure within the PowerMill standard library for the corresponding ABB robot manufacturer.

**Figure 5:**
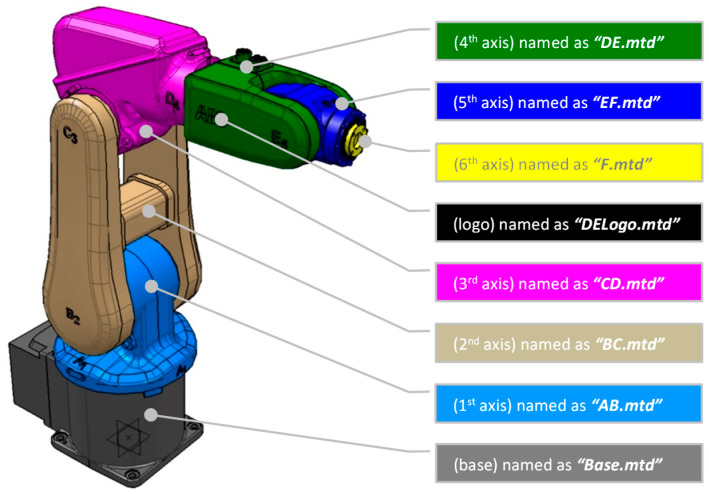
Naming the robot mtd CAD files.

**Figure 6:**
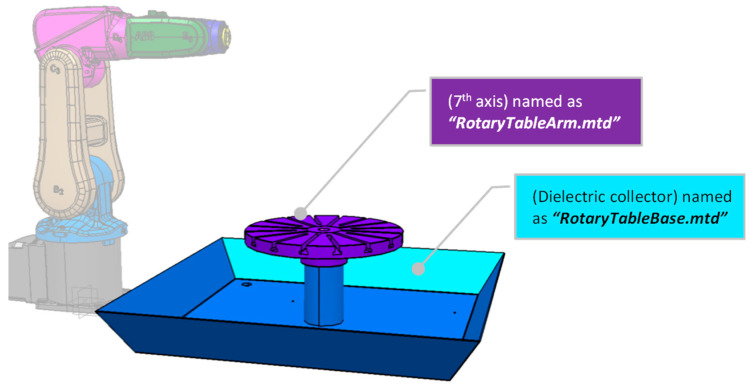
Naming the mtd CAD files for the 7th axis (rotating table) and dielectric collector.

To build the DT, PowerMill uses a specific CAD extension file of the mtd type. PowerMill itself can be used to convert the prepared STEP files. Thus, the second step is to import the step files individually and double-check their position and orientations so they can be re-exported as converted mtd files within the created folder “ABB-IRB120”, with the specific file names shown in Figure 5 and Figure 6, respectively. Similarly, the end-effectors, the 7th axis, and the dielectric collector will be converted to mtd-type files to be saved in the same folder.

The next step is to create an mtd file structure in the xml code format that describes all the specific information necessary to control the robot simulation using PowerMill. Using Notepad++ v8, the following variables were adjusted: path for the 3D mtd folder, milling and wire EDM end-effector attachment points and orientations, 6th axis head attachment point coordinates and orientation, the rotating table attachment point coordinates and orientation, the axis minimum and maximum angle values, the robot home position, and the rotation center.

**Figure 7:**
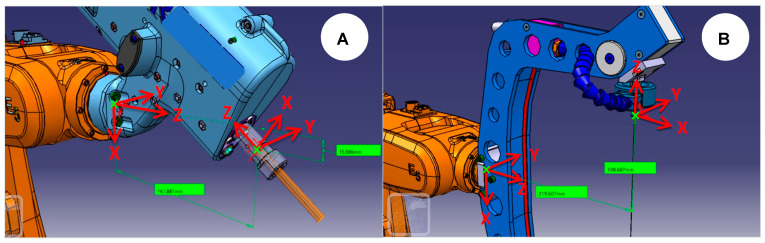
MEDM (**A**) and WEDM (**B**) electrode tool workplane for offline programming using PowerMill.

At this point, the orientation and position have been taken relative to the world origin coordinates *XYZ* (0,0,0). However, the end-effector uses a relative origin of the 6th axis attachment point, where displacement and reorientations will be necessary to depict each EDM end-effector’s electrode tool workplane and attachment point. Thus, we first move the attachment point along the *X*, *Y*, and *Z* axes using the 6th axis workplane as the reference. Next, we rotate *Z*, *Y*, and *X* around the transformed workplane to obtain the resulting Euler orientation. As a result, Figure 7 depicts the workplane transformation for the milling EDM end-effector (MEDM) and wire EDM end-effector (MEDM).

After adjusting the mtd files, the DT is ready and can be used for MEDM and WEDM by swapping the end-effector model name, followed by a new electrode attachment point and orientation, as shown in Figure 7.

### 3.6. Calibration of the Robotic EDM Cell

Even high-stiffness structures made using traditional 3-axis CNC can incur 21 types of mechanical translation and rotational errors of ±20 μm, even after calibration [31]. Similarly, IRs must cope with manufacturing and other errors caused by payload deflection and backlash. However, unlike conventional machining, EDM must sustain a discharge gap of about 20 μm. Therefore, significant calibration errors can potentially compromise the spark gap, and the EDM process will not be performed either by lack of sparkles or short circuits. In our case, building and commissioning the EDM robot apparatus revealed that the physical robotic cell assembly will always have inherent manufacturing inaccuracies compared to the ideal and virtually free-of-error CAD robotic cell. Thus, the present study focuses on achieving EDM-feasible and accurate cutting programs, optimizing the triad of (i) the physical robotic cell, (ii) the real robot controller coordinate and orientation system, and (iii) the three-dimensional CAD models used to create a novel digital twin simulation and offline programming that ensures thoughtful calibration convergence.

**Figure 8:**
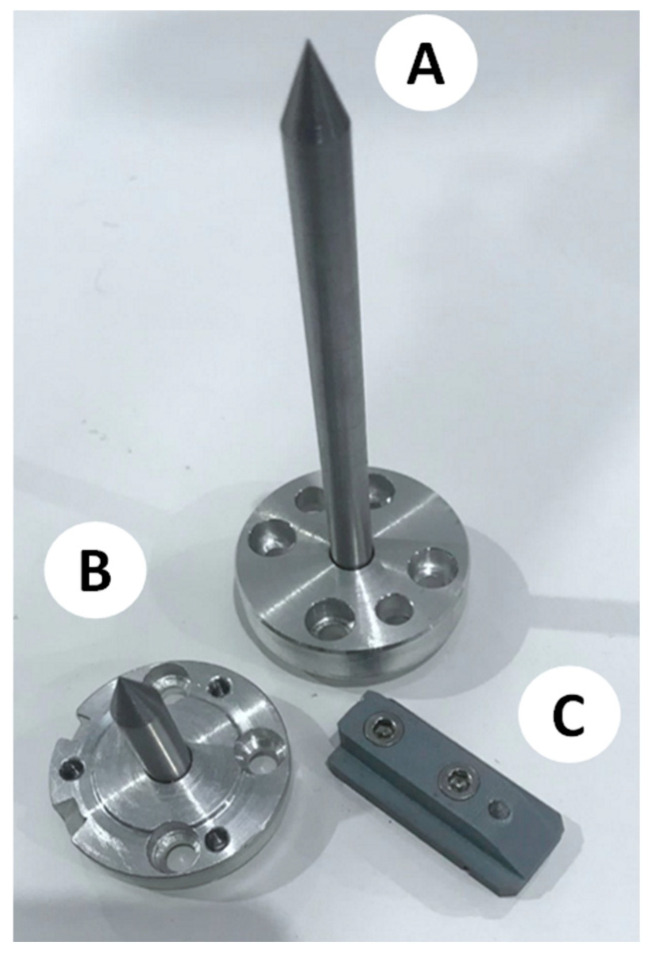
Robotic EDM cell calibration tools (A: Long probe, B: Short probe, C: Table probe).

Therefore, the calibration procedure is performed in two steps. First, the robotic cell is calibrated. In other words, the 6-axis robot arm and the 7-axis rotating table will be calibrated to recognize each other. The procedure will identify and quantify the geometric and positional errors to calibrate the ABB robot controller so that offline robot programming can automatically compensate for the errors. Next, each end-effector will be analyzed to consider its manufacturing errors and assembly and deflection errors caused by its specific payload. To move forward, the set of calibrated probes shown in Figure 8 is necessary to execute both robot cell and end-effector calibration. This is explained below.

**Figure 9:**
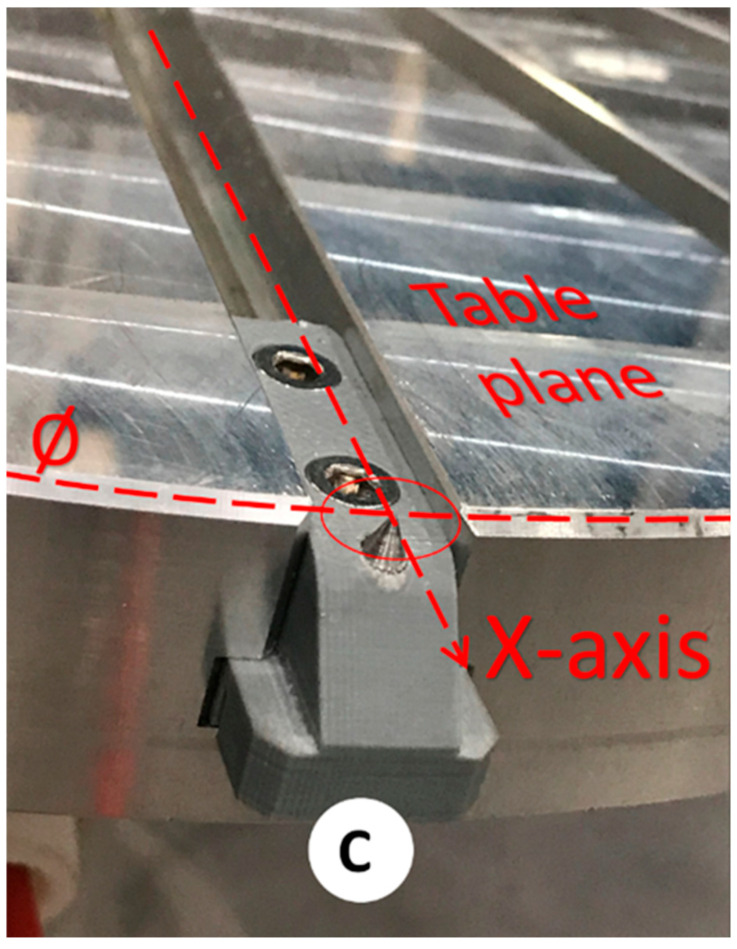
The 7th axis rotating table calibration: probe C installation.

- Two long probes (Figure 8A) of Ø8 mm × 147.22 mm length are used for cell and end-effector calibration. Each of them comprises a central rod and a flange to allow for its direct assembly on the robot’s 6th axis.
- A short probe (Figure 8B) of Ø8 mm × 67.65 mm in length is used for cell and end-effector calibration. This probe is precisely the same as the long probe except for its length.
- A table probe (Figure 8C) composed of a 3D-printed body and an inserted metallic probe is designed to be assembled in the holding slots of the rotating table so that the tip of the probe can capture the table’s diameter, surface (working plane), and *X*-axis. Figure 9 presents the calibration assembly setup of probe C on the 7th axis.

**Figure 10:**
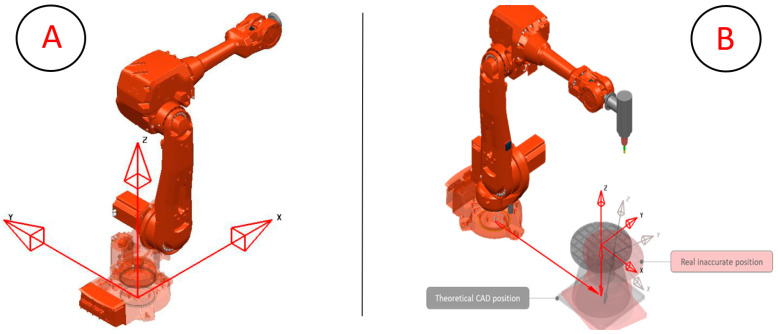
(**A**) ABB world coordinates and orientation. (**B**) Theoretical vs. actual position of the 7th axis.

The calibration process relies on the assumption that the robot is correct and calibrated so that it defines the world origin coordinates *X*, *Y*, and *Z* located in the center of the robot base. Since not all robot manufacturers use the same coordinate system, Figure 10A depicts the standard robot orientation for the adopted ABB robot. Therefore, calibrating the cell requires identifying where the 7th-axis rotating table is located, including its positional and orientation errors, as shown in Figure 10B.

**Figure 11:**
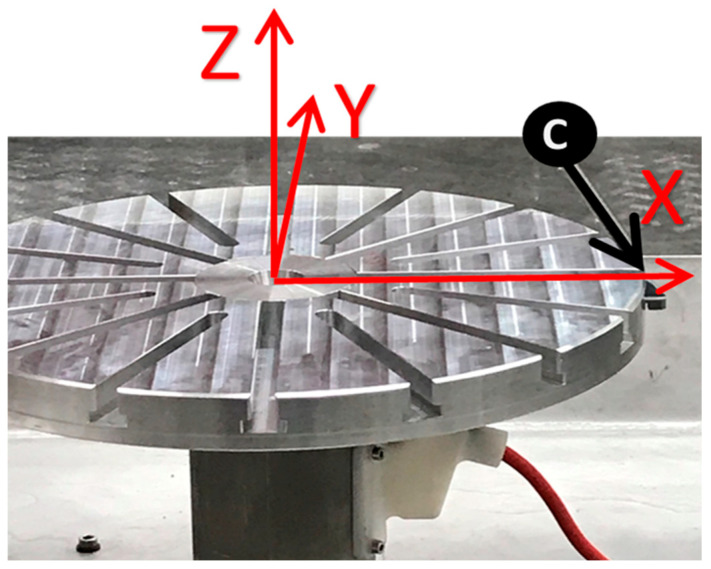
Table probe C with the rotating table at 0°.

**Figure 12:**
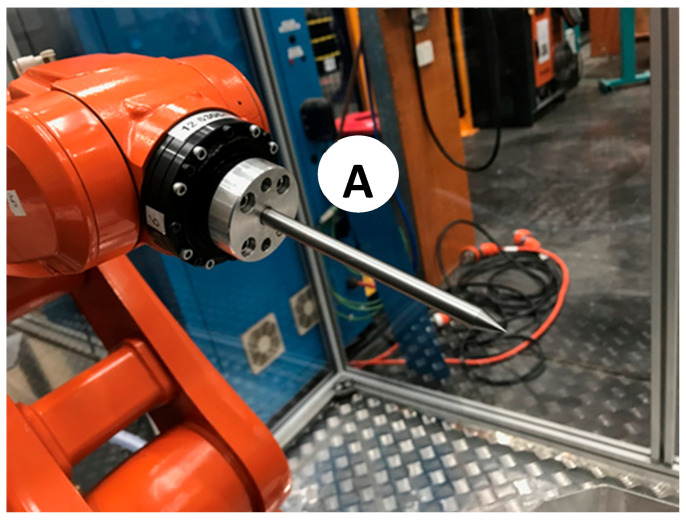
Calibration long probes A are used as end-effectors in the 6th axis of the robot.

**Figure 13:**
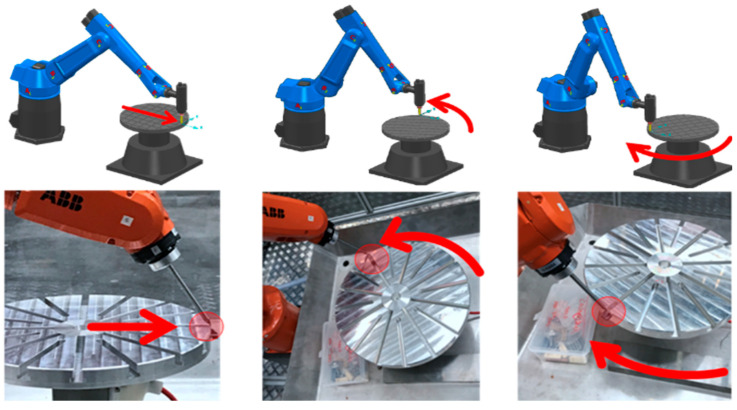
Rotating table center calibration.

The robot cell calibration procedure must be performed once after cell installation. However, it must be repeated after assembly changes or collisions. The aim of the cell calibration is (i) to define the table center point coordinates (*XYZ*) from the robot’s world plane, (ii) to define the table’s work surface by the *XY* plane, and (iii) to define the table *X* and *Z* axis directions, which can be achieved using the following steps:

1. First, the table is rotated at 0° to next assemble the table probe in the table slot, which theoretically coincides with the *X*-axis of the robot. Figure 11 shows the result.
2. Next, the long probe is assembled on the robot’s 6th axis as an end-effector, and its tip depicts the TCP so that the robot becomes a 3D measurement tool. Figure 12 shows the result.
3. Three different points in space can define a circle and its plane. Thus, to find the center of the rotating table and its working plan, three coordinates (XYZ) of three unique position points are measured. To find the three points, the table is rotated in intervals of 120°, starting at 60°, 180°, and 300°, aiming for them to be as far as possible from each other to enhance accuracy. Next, the robot is manually positioned so that the tip of the long probe is as close as possible to the tip of the table probe. Once the circle is found, it defines the central point of the rotating table and its supporting plan. Figure 13 summarizes this step.
4. Next, the table is rotated back to 0°, and a new measurement of the Cartesian coordinates (*XYZ*) for the fourth point is acquired, which, together with the table center point, will define the *X*-axis table orientation.
5. Using PowerMill, each Cartesian coordinate *X*, *Y*, and *Z* of the four acquired points is used to calculate the actual position and orientation of the 7th axis to find the real center of the rotating table, as well as the table plan orientation.
6. Finally, there are three final tasks to achieve an updated system that matches the detected errors. They consist of updating the identified deviations into the DT and physical systems involved, meaning (i) the ABB robot controller table center and orientation are updated, and (ii) the digital twin 3D CAD files modeled in the 3D CAD system CATIA are moved to reflect the errors, to then be re-exported, converted, and updated as previously described in Section 3.3, Section 3.4 and Section 3.5. Lastly, (iii) the PowerMill xml code files, responsible for defining the position, orientation, and rotation axes of the updated 3D CAD files, are updated.

### 3.7. Calibration of the EDM End-Effectors

Once the robotic cell with the 7th axis is calibrated, it is necessary to calibrate the end-effectors. To do so, the ABB 4-point calibration method is adopted and repeated three times for each probe so that the resulting accuracy can be approximated to that of CNC machines [32].

**Figure 14:**
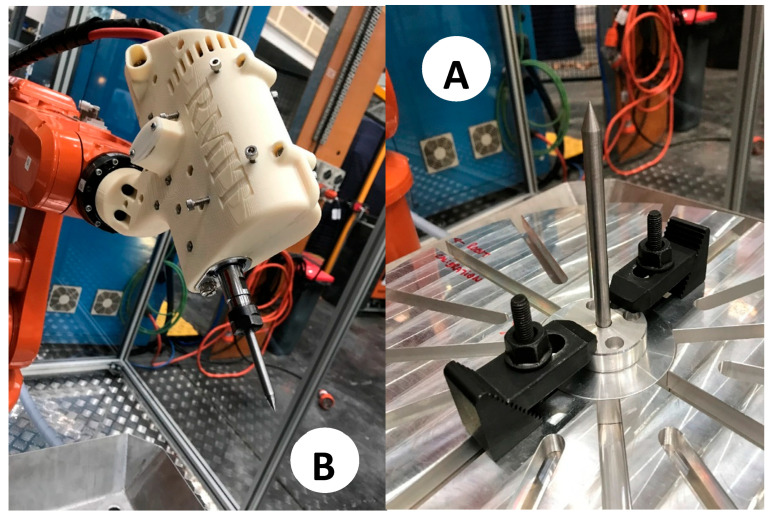
MEDM end-effector calibration. (**A**): Fixed long probe, (**B**): End-effector long probe.

Figure 14 presents the installed MEDM end-effector using the short probe B where the electrode tool would be. At the same time, the long probe A is fixed over the calibrated plane XY of the rotating table. It is worth noting that the long probe A works as an aleatory yet fixed reference while the table remains static. The only criteria for locating the long probe A are that the robot can reach it in a significantly different pose, which is, in our case, strategically designed to be the center of the rotating table.

The 4-point method finds the Tool Coordinate Point (TCP) of the short probe, the relative position of the robot base on axes X, Y, and Z, and its rotational position [Q_1_, Q_2_, Q_3_, Q_4_] based on quaternions from a rotational matrix [33] in Equation (1).

(1) $$\left[\begin{array}{ccc} x_{1} & y_{1} & z_{1}\\ x_{2} & y_{2} & z_{2}\\ x_{3} & y_{3} & z_{3} \end{array}\right]⟺\left[\begin{array}{cc} Q_{1}=\frac{\sqrt{x_{1}+y_{2}+z_{3}+1}}{2} & \\ Q_{2}=\frac{\sqrt{x_{1}-y_{2}-z_{3}+1}}{2}, & sign\;Q_{2}=sign\left(y_{3}-z_{2}\right)\\ Q_{3}=\frac{\sqrt{y_{2}-x_{1}-z_{3}+1}}{2}, & sign\;Q_{3}=sign\left(z_{1}-x_{3}\right)\\ Q_{4}=\frac{\sqrt{z_{3}-x_{1}-y_{2}+1}}{2}, & sign\;Q_{4}=sign\left(x_{2}-y_{1}\right) \end{array}\right]$$

**Figure 15:**
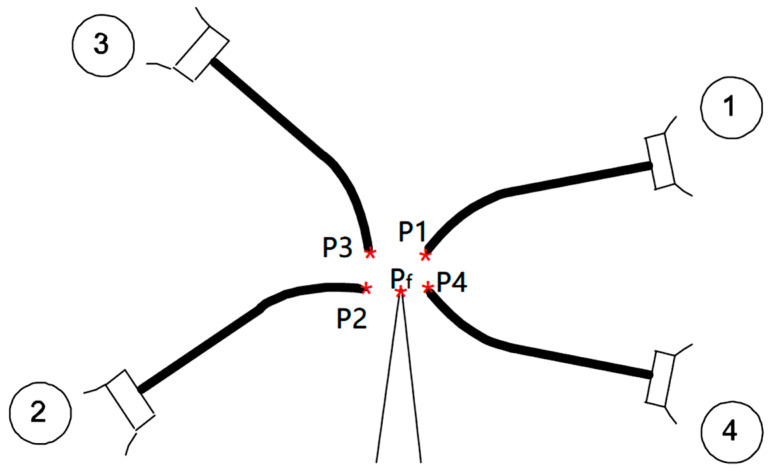
Four-point end-effector calibration method.

Since the short probe is visually positioned by manually moving the robot, a human cannot approach the exact position as the fixed long probe. Thus, the four-point coordinates *P_Tx_B*, *P_Ty_B*, and *P_Tz_B* are different in each of the four robotic poses in Figure 15.

Therefore, the TCP of the four points can be calculated by Equation (2).

(2) nxoxaxnyoyaynzozaz.PTxEPTyEPTyE+PExBPEyBPEzB=PTxBPTyBPTzB

Since the 4-point method is manual and visual, there will be inherent human errors. Thus, the *P_f_* theoretical position vector needs to be calculated by averaging the position vectors of *P*_1_, *P*_2_, *P*_3_, and *P*_4_ as in Equation (3), while *P_f_* is the fixed tip of the long probe (Figure 15).

(3) $$P_{f}=\frac{1}{n}\left(\sum_{i}^{n}P_{i}\right)=\frac{P_{1}+P_{2}+\cdots P_{n}}{n}$$

Once the position vectors of *P_f_* are identified, the distance d_i_ between *P_f_* and *P_i_* can be calculated by Equation (4).

(4) $$d_{i}=\sqrt{{\left(P_{fx}-P_{ix}\right)}^{2}+{\left(P_{fy}-P_{iy}\right)}^{2}{+\left(P_{fz}-P_{iz}\right)}^{2}}$$

After obtaining the distances between *P_f_* and each point *P_i_*, the mean error from the calculated TCP is the mean value of these distances, as in Equation (5).

(5) $$Mean\;error\;from\;the\;calculated\;TCP=\frac{1}{n}\left(\sum_{i}^{n}d_{i}\right)=\frac{d_{1}+d_{2}+\cdots d_{n}}{n}$$

Finally, aiming to detect discrepancies in manually acquiring the four points, the maximum error from the calculated TCP is the maximum value of these distances by repeating the calculations using Equation (4).

According to the robot manufacturer, to achieve the robot’s specified accuracy of 0.01 mm, the process must be repeated whenever the error discrepancy is superior to 0.4 mm [33]. In our case, the process was repeated two times, finding an error not superior to 0.27 mm. The 4-point method to calibrate the exact tool *N* times will not deliver the same accuracy. The main reason is that the 4-point process is visual, so the operator will always introduce some error that may compromise the EDM process. Thus, to further improve the calibration to the point that the robot performance approximates a CNC machine, the PowerMill 3-point spindle calibration method is adopted. The objective of the additional calibration is to overcome visual calibration errors and increase accuracy about (i) the spindle center point (*XYZ*) and (ii) the spindle direction (*IJK*), which defines the tool axis.

**Figure 16:**
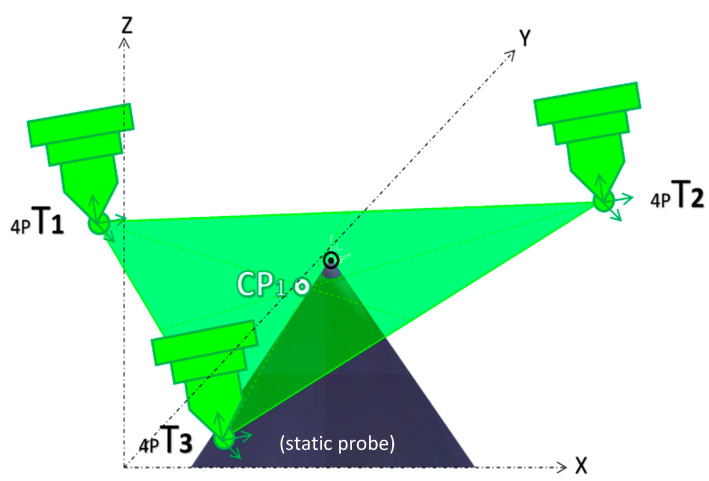
Three-point averaged error for the short probe, CP_1_.

**Figure 17:**
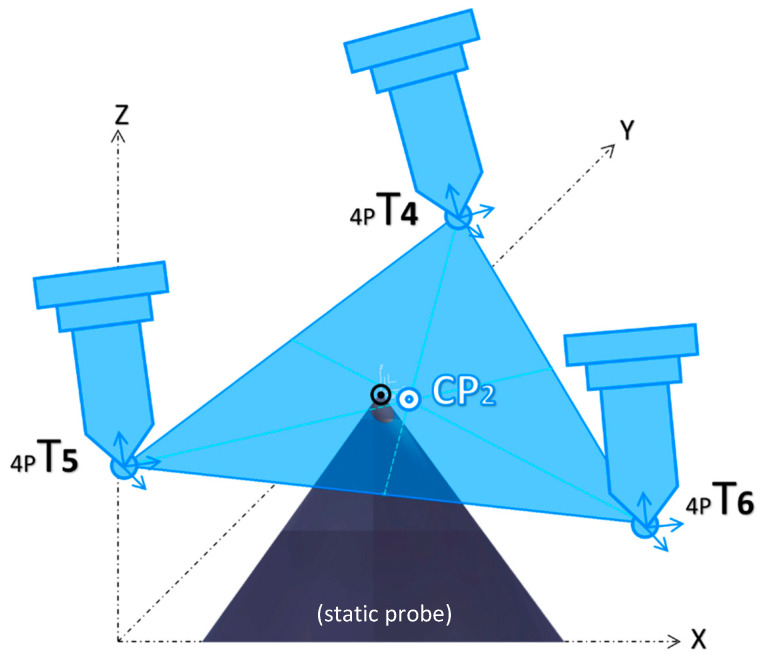
Three-point averaged error for the long probe, CP_2_.

The PowerMill spindle calibration method can be considered an extension of the 4-point method. Within the PowerMill, the 4-point method is referred to as teaching, and it is used to capture the *X*, *Y*, and *Z* coordinates of each teaching point *4pT*. The 4-point process is repeated three times with a long and short probe, while a third long probe installed at the center of the rotating table is kept fixed. Once the three teaching points of each short and long probe are found, they are used to create two triangles. Next, to average visual errors, it is assumed that each triangle centroid *CP_X,Y,Z_* is the best approximation of the respective probe length. The result of this step is two centroid points, *CP*_1_ and *CP*_2_, calculated according to Equation (6) and depicted in Figure 16 and Figure 17, respectively.

(6) $${CP}_{X,Y,Z}=\left(\frac{X_{{4pT}_{1}}+Y_{{4pT}_{1}}+Z_{{4pT}_{1}}}{3},\frac{X_{{4pT}_{2}}+Y_{{4pT}_{2}}+Z_{{4pT}_{2}}}{3},\frac{X_{{4pT}_{3}}+Y_{{4pT}_{3}}+Z_{{4pT}_{3}}}{3}\right)$$

**Figure 18:**
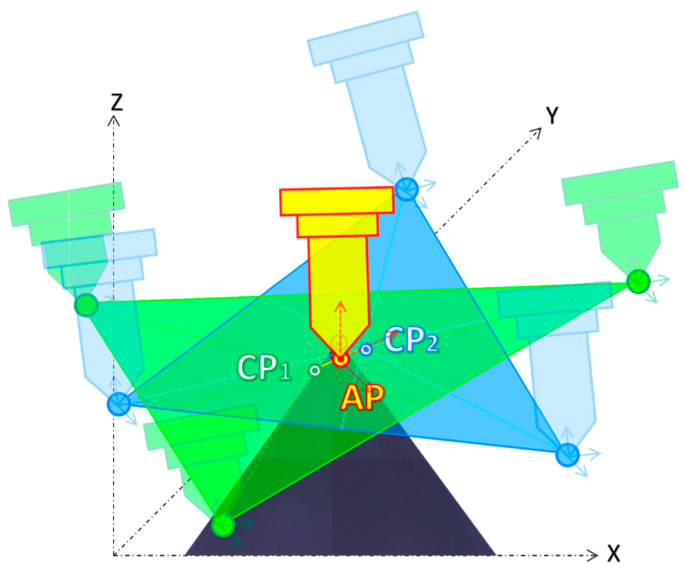
Line segment by *CP*_1_ and *CP*_2_.

The final coordinates of the error-averaged point AP are given by the midpoint of the line segment, defined by the endpoint of *CP*_1*(X,Y,Z)*_ and *CP*_2*(X,Y,Z*)_, as in Equation (7), while Figure 18 demonstrates how significantly improved the new calibration of point AP is. Hence, the same segment $\bar{{CP}_{1}{CP}_{2}}$ defines the tool axis vector (*I*, *J*, *K*).

(7) $${CP}_{X,Y,Z}=\left(\frac{X_{{CP}_{1}}+X_{{CP}_{2}}}{2},\frac{Y_{{CP}_{1}}+Y_{{CP}_{2}}}{2},\frac{Z_{{CP}_{1}}+Z_{{CP}_{2}}}{2}\right)$$

Using PowerMill, the teaching point inputs and the results of the MEDM end-effector are found, followed by the “head attachment point” to update the DT. Lastly, calibrating the WEDM end-effector follows the same procedures, except that the wire substitutes for the end-effector probes. Thus, two points at 1/3 and 2/3 of the tensioned wire are marked and considered the tips of the short and long probes, respectively.

**Figure 19:**
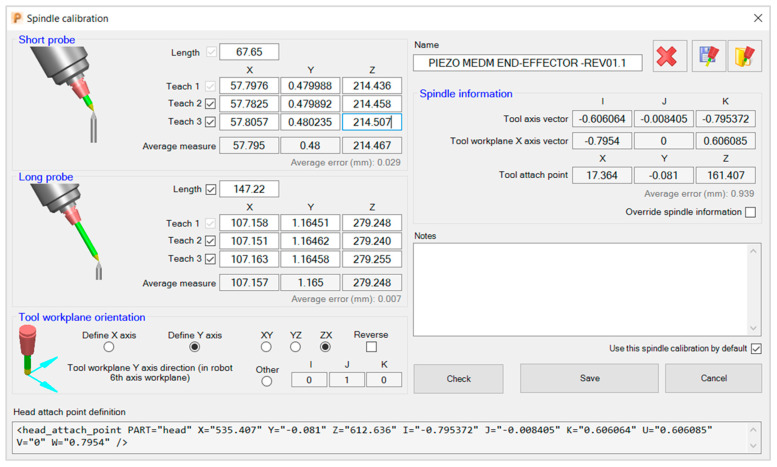
PowerMill MEDM end-effector calibration interface.

**Figure 20:**
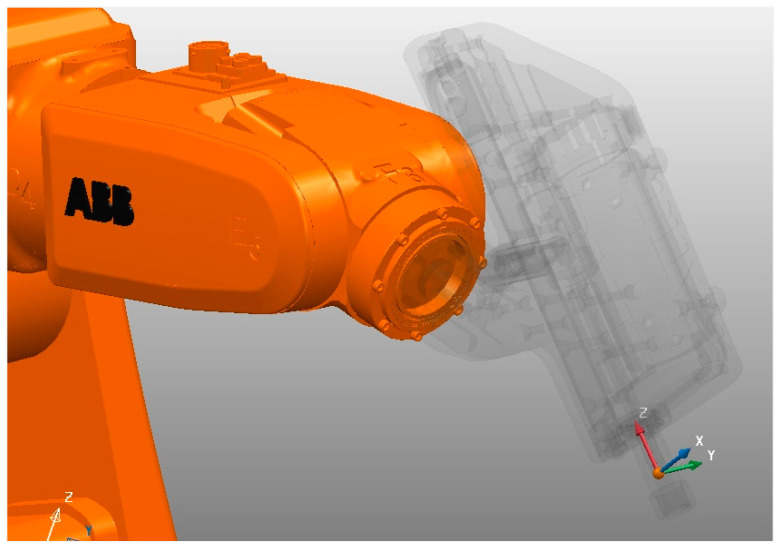
PowerMill MEDM end-effector (spindle) calibration.

Finally, the PowerMill interface in Figure 19 presents the teaching point inputs and the results of the milling EDM end-effector. Also, a “head attachment point” line code is generated to be updated within the virtual robotic cell for accurate DT simulation and offline programming xml files; its ultimate result is a well-calibrated MEDM end-effector, as shown in Figure 20.

## 4. Validation Results

In this section, we conduct two experimental evaluations of the robotic EDM digital twin’s capabilities to confirm both its (i) gap accuracy and (ii) cutting path programming.

**Figure 21:**
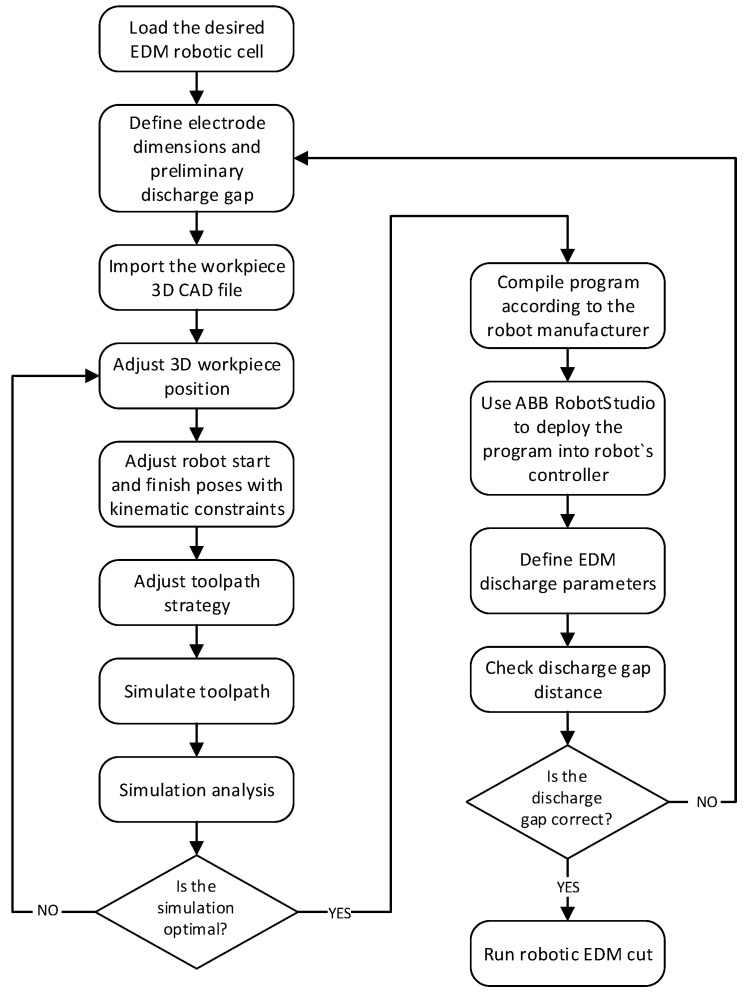
Robotic EDM DT programming workflow.

Once the DT of the robotic cell is adequately built and calibrated, Figure 21 presents the detailed steps for offline programming using the developed digital twin application and its experimental validation.

### 4.1. EDM Cutting Path Gap Accuracy

First, it is paramount to confirm the calibration and accuracy capabilities of the robotic EDM digital twin. The MEDM end-effector is selected for this end, while the critical EDM process parameter of gap distance is the variable of interest. To play the role of the workpiece, a gauge block (DIN 861-1980 grade A) with a parallelism and flatness tolerance of +0.15 μm to −0.05 μm is adopted. Next, a CAD version of the gauge block is modeled, while the physical sample is assembled on the seventh-axis table. Finally, we adopt a helical path from the PowerMill cutting path library. In the program, the pulse gap distance is set to approach and stop the electrode parallel to the block at a 50 µm gap distance.

**Figure 22:**
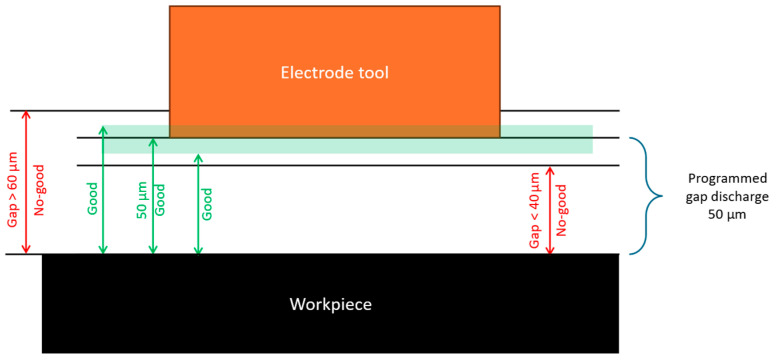
Expected contact regarding the programmed path.

**Figure 23:**
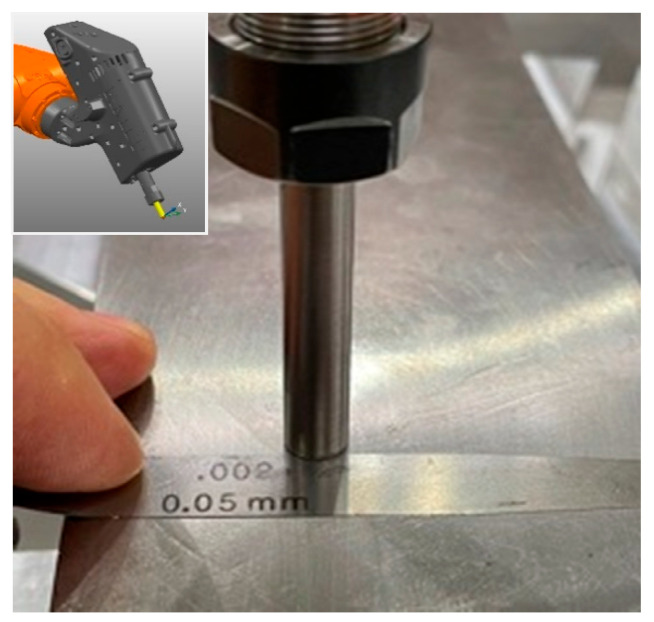
Verification of calibrated MEDM pulse gap distance.

The experiment validation adopts the Central Limit Theorem [34], which states that the sampling distribution of the mean of a sufficiently large sample size approximates normality, regardless of the shape of the population distribution. Thus, the program is executed 200 times, 100 with the robot approaching the workpiece starting from the home position and the other 100 from random positions to mimic the restart of the programs or new path trajectories in actual cutting conditions. To measure the gap, three stainless-steel shims of 60 µm, 50 µm, and 40 µm are used as a no-go gauge to verify the accuracy of the programmed gap. The use of the shim technique is particularly useful because it evaluates the gap not just as a point-by-point evaluation but also allows one to grasp how good the parallelism between the tool and the workpiece is. The attribute criteria for good and no-good are depicted in Table 1, while Figure 22 demonstrates the gap criteria and Figure 23 shows the real evaluation.

### 4.2. Experimental Validation of Offline Cutting Path Programming Using the DT 

**Figure 24:**
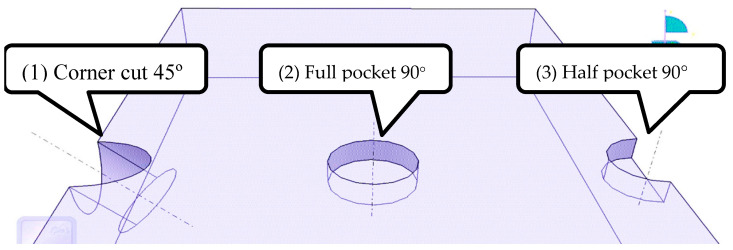
Experimental MEDM workpiece pocket approaches.

Once the EDM calibration is proven adequate, a cutting path is generated and deployed to the physical twin. The chosen geometry to be machined is a circular pocket of 12 mm in diameter, varying in depth, position, and angle. Figure 24 depicts the three experimental approaches.

**Figure 25:**
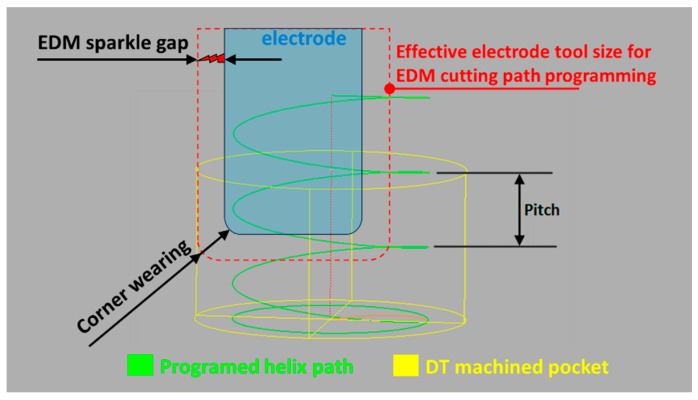
Helical cutting path strategy for milling EDM.

The adopted cutting strategy is a helical path with a pitch defined as 0.01 mm. Next, to anticipate the EDM wear of the electrode corner, the resulting radius is defined as 0.2 mm by approximating the cutting pitch as in Ding and Jiang [35]. Figure 25 depicts the cutting path strategy, embedding an EDM discharge gap and offline wear compensation for electrode length and radius corner. In contrast, Figure 1 compares and confirms the adequate reproduction of both the DT and the robotic EDM physical object.

**Figure 26:**
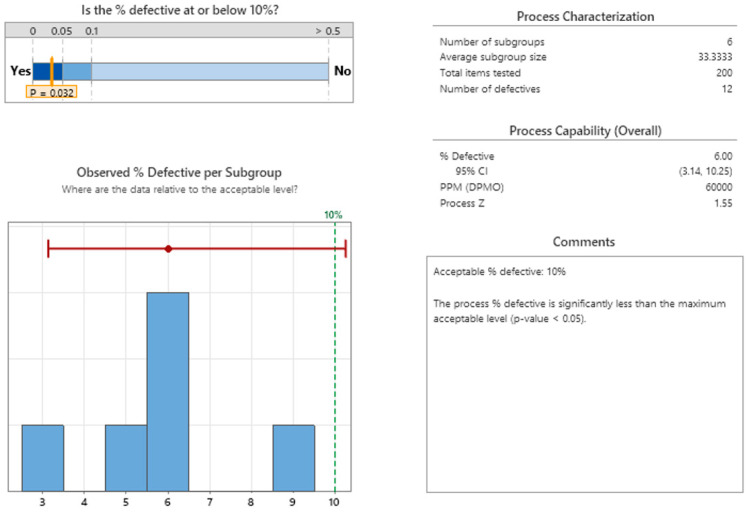
Binomial capability: summary analysis of EDM gap.

**Figure 27:**
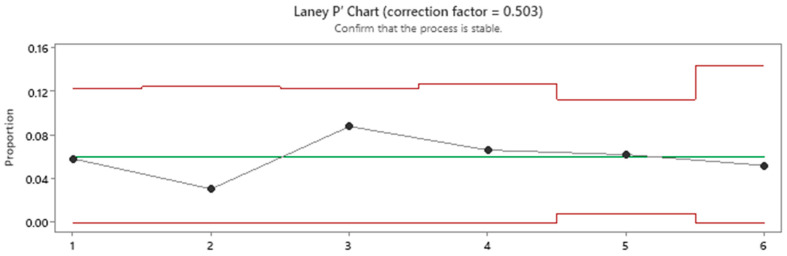
Laney P’Chart for gap stability.

**Figure 28:**
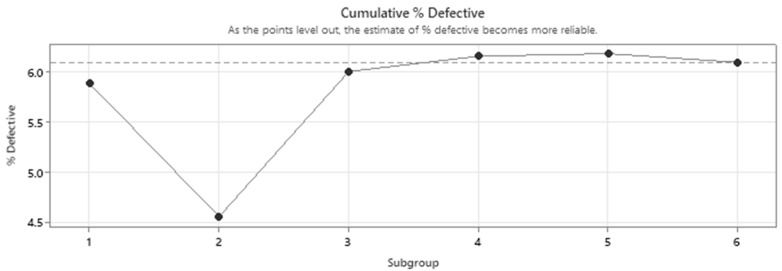
Cumulative % of defective discharge gaps.

To verify the experimental results, we evaluate the capability of the process in sustaining a regular gap discharge. To that end, we used Minitab v22.1 to perform a binomial capability analysis, collecting data from six different sub-group batches and a target of no-good gaps limited to 10%. An example of the measuring procedure is given in Figure 26, while Figure 26, Figure 27 and Figure 28 present the capability results.

Despite the limited sample size in the analysis, with six subgroups, the 10% gap error objective was achieved. Hence, using the 95% confidence interval for the 10% target of defective gaps revealed that a smaller target of approximately 6% could be achieved.

As per Figure 27, the proportion of gaps is stable and no points are found to be out of control (out or red lines), suggesting a stable process.

The cumulative defects (Figure 28) depict a stable index of defective gaps, due to which the EDM discharge process can be interpreted as feasible.

## 5. Conclusions

**Figure 29:**
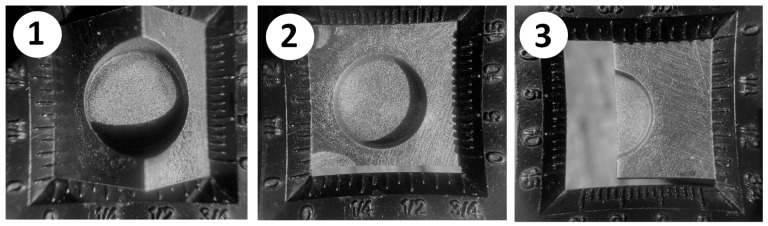
(**1**) Corner cut 45°, (**2**) full pocket 90°, (**3**) half pocket 90°.

Due to the novelty of multi-axis robotic machining based on electric discharge machining (EDM), current CAD/CAM systems lack automated cutting path process planning capabilities that fulfill specific EDM needs regarding the accuracy of discharge gaps, together with the generation of complex, seven-axis paths. To fill this research gap, this paper presented a robust step-by-step method to develop, calibrate, and validate a robotic EDM digital twin created within PowerMill, a mature and well-accepted toolpath CAM system. The findings confirm that importing and converting any 3D workpiece models into cutting paths for complex seven-axis robotic trajectories is now possible and practical. The experiments, conducted with statistical relevance, demonstrated that the critical EDM gap distance has an accurate variation of no more than 20 μm, followed by a stable repeatability of the robot, demonstrating a feasible discharge gap for the EDM process. Furthermore, the DT proved valuable for conducting kinematic simulations to detect and avoid collisions and robotic singularities, which are aspects of utmost importance in multi-axis operations. Finally, the offline programming became CNC-like and practical, so that the currently trained academic or industrial workforce, familiar with the PowerMill CAM software interface, can easily reuse the proposed DT. Lastly, the generated path code was successfully exported in ABB’s native robotic language while allowing for the reuse of mature and broadly accepted cutting path machining techniques. It is worth noting that EDM experiments, including a proper EDM controller, were conducted with the generated cutting path and proved feasible, as shown in Figure 29. However, due to the complexity of EDM control and all the needed evaluations, a specific paper was written to properly cover many aspects, including EDM control, robotic vibration, electrode wear, machined accuracy, and surface roughness [3].

Finally, since data between physical and digital objects is exchanged manually, changes in the physical object’s state are not directly reflected in the digital one, and vice versa. Therefore, based on the full capabilities of sophisticated digital twins, the proposed solution falls into the Digital Model type, and establishing automatic signal communication between the twins to close the loop and further optimize the EDM process is left to future research.

## Figures and Tables

**Table 1:** Attribute analysis on the gap discharge of the robotic EDM cutting path gap.

Inspection Scenario	Gauge Thickness for Contact Detection			Considered Gap Interval (μm)	Attribute Result	
	40 μm	50 μm	60 μm		Good	No-Good
Scenario 1	no	no	no	g > 60	44	
Scenario 2	yes	no	no	50 < g < 60		1
Scenario 3	yes	yes	no	40 < g < 50	43	
Scenario 4	yes	yes	yes	g < 40		2

## Data Availability

The authors do not have permission to share the data.
